# Use of fibrin glue in periodontal flap surgery

**DOI:** 10.4103/0972-124X.44094

**Published:** 2008

**Authors:** Bimal Jathal, Anal Trivedi, Neeta Bhavsar

**Affiliations:** 1 *Dean, Faculty of Dental Sciences and Head of the Department of Periodontia, Dharmsinh Desai University, Nadiad – 387 001, Gujarat, India*; 2 *Lecturer, Department of Periodontology, Faculty of Dental Sciences, Dharmsinh Desai University, Nadiad – 387 001, Gujarat, India*; 3 *Professor, Department of Periodontology, Government Dental College and Hospital, Ahmedabad – 380 016, India*

**Keywords:** Fibrin glue, sutureless periodontal surgery, periodontal flap closure

## Abstract

In the changing era of perio surgeries one innovative remedy has ended the inconvenience of suturing and has allowed the clinician to meet growing expectations and demands of today's dental patient, and the remedy is fibrin glue. When periodontal plastic surgical procedures done or implants placed in esthetic zone, fibrin sealants may be variable alternative to closing flaps with sutures and with histologic benefits and has potential uses in field of medicine. Fibrin sealant is an excellent beginner in the era of sutureless periodontal flap surgery, and this article is a humble effort to prove it.

## INTRODUCTION

### What is fibrin glue

Fibrin glue is "Fibrin Fibronectin Sealing System (FFSS)". It is available as two component system: first component contains highly concentrated fibrinogen, factor XIII, fibronectin, and traces of other plasma proteins. The second component contains thrombin, calcium chloride, and antifibrinolytic agents such as aprotinin. Mixing of two components promotes clotting with the formation and cross-linking of fibrin.

**Figure 1:**
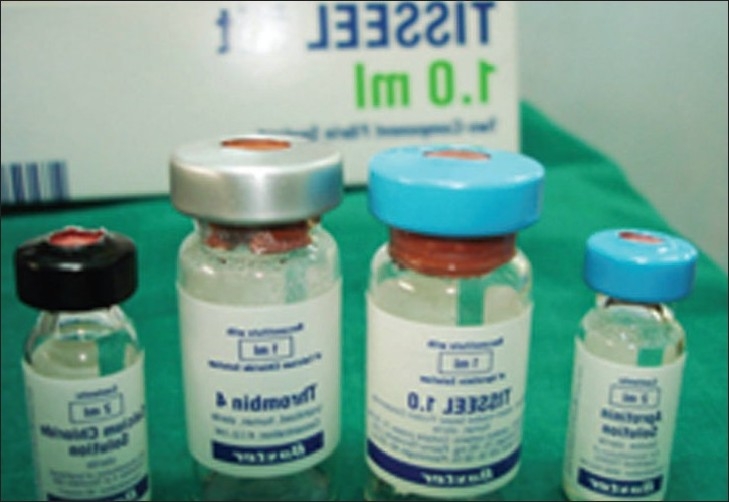
Fibrin glue kit

Fibrin glue is commercially available as Tisseel VH (Baxter, U.S.A.) and Tissucol (Termo trattato,Wien.) [Figure 1].

### Applications of fibrin glue in dentistry

1. Fibrin-sealing system is effective as a means of fixing tissues after periodontal surgery, as fibrin glue is easier and quicker to use than sutures. Sutures cause inflammation around themselves, while fibrin glue enhances early wound healing. In periodontal plastic surgeries of esthetically impo*r*tant areas it gives better results than sutures.
2. The split mouth clinical trials done to see effect of treating deep wide buccal gingival recession with guided tissues regeneration procedure after root conditioning with tetracycline HCI and FFSS give good results.
3. Recent animal studies showed that FFSS has osteoconductive potential and significantly produced more new bone and new connective tissue when used with bone graft material like β-tricalcium phosphate

### Applications in field of medicine

1. Fibrin sealant is indicated for use as an adjunct to hemostasis in surgeries involving cardiopulmonary bypass and treatment of splenic injuries due to blunt or penetrating trauma to the abdomen.
2. Fibrin sealant has been shown to be an adjunct in closure of colostomies.
3. Fibrin sealant is a satisfactory hemostatic agent in fully heparinized patients undergoing cardiopulmonary bypass.

### Method of preparation and application

Nowadays fibrin sealant kit contains four separate vials: (1) sealer protein concentrate (human) vapor heated, freeze dried; (2) fibrinolysis inhibitor solution (bovine); (3) thrombin (human) vapor heated freeze dried; and (4) calcium chloride solution.

Freeze dried sealer protein concentrate and thrombin are reconstituted in fibrinolysis inhibitor solution and calcium chloride solution, respectively, by using fibrinotherm heating and stirring device, a standard incubator or a water bath.

**Figure 2:**
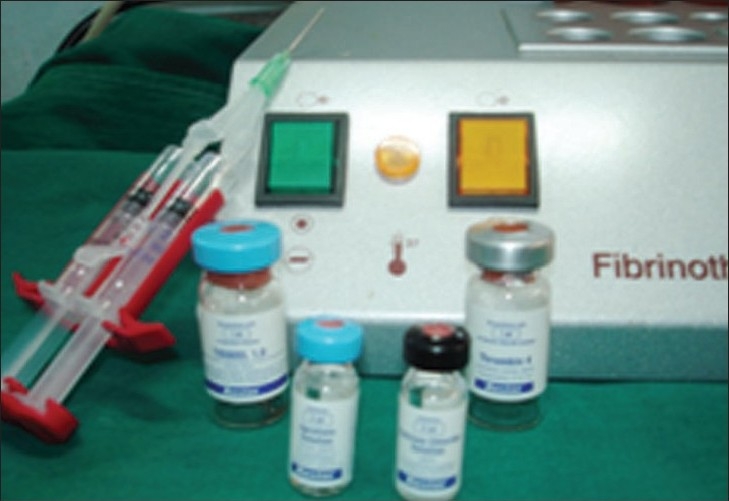
Fibrin glue kit with fibrinotherm

**Figure 3:**
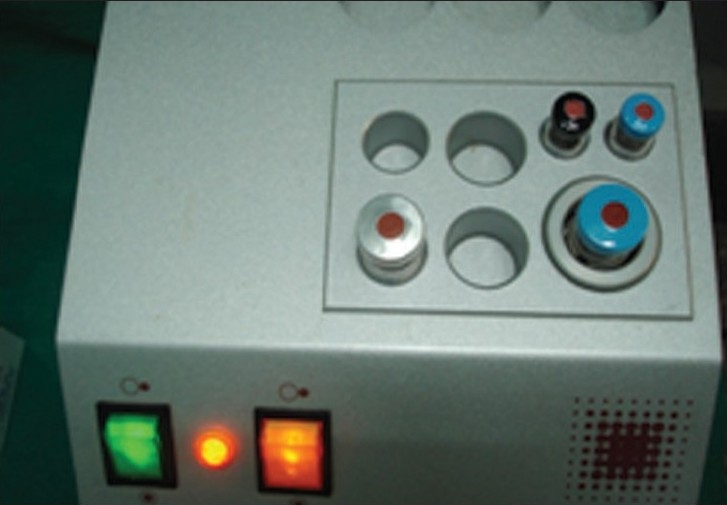
Fibrinotherm heating and duploject syringe and stirring device

**Figure 4:**
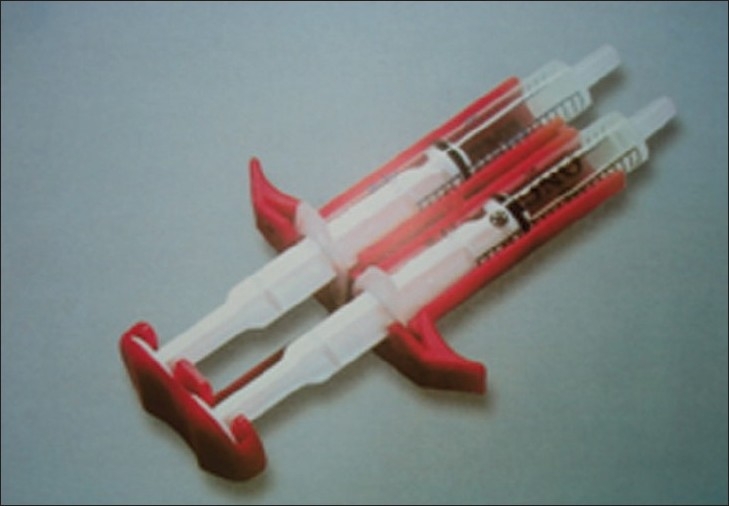
Duploject syringe

**Figure 5:**
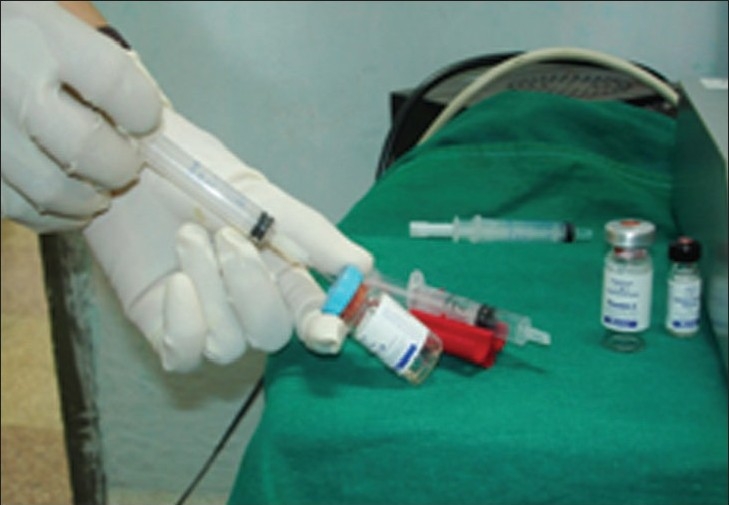
Filling of duploject syringes

Solutions are then combined by using duploject and applied on average 0.1 ml per tooth on each side (0.2 ml in cases of flap procedure on both buccal and lingual aspects) [Figures 2–5]).

### Precautions and limitations

1. Fibrin sealant cannot be used in individuals who are known to be hypersensitive to bovine protein.
2. Fibrin sealant cannot be indicated for the treatment of massive and brisk arterial or venous bleeding.
3. To avoid risk of allergic anaphylactic reaction and/or thromboembolic events, which may be life threatening, fibrin sealant should not be applied intravascularly or into the tissues.

## CASE REPORTS

Here, we report of two patients in whom flaps were closed using fibrin in the first patient and sutures in the second. The aim was to check the consequence of fibrin sealant as an alternative to sutures [Figures 6–17]. There was a definite ease of usage on the part of clinician of the fibrin glue, while there was painless and early recovery of the glued area in the first patient as compared to the sutured area in the second patient.

**Figure 6:**
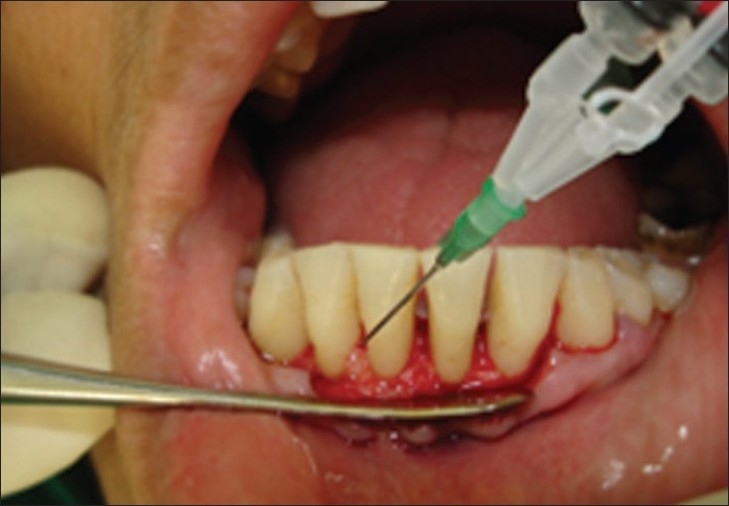
Fibrin glue application; Case 1: After debridement, fibrin glue being applied on bone and both facial and lingual flaps followed by flap approximation for 3 minutes under finger pressure

**Figures 7 and 8:**
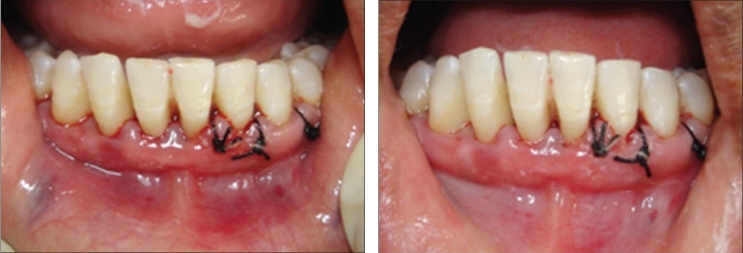
Right side flap approximation with fibrin glue is compared with sutures on left side. Note the stoppage of bleeding immediately after 30 seconds on fibrin glue side

**Figure 9:**
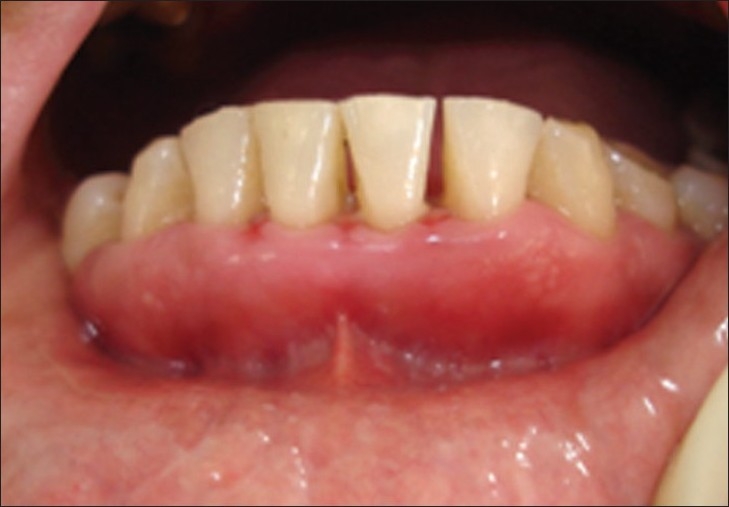
After 3 days, case 1

**Figure 10:**
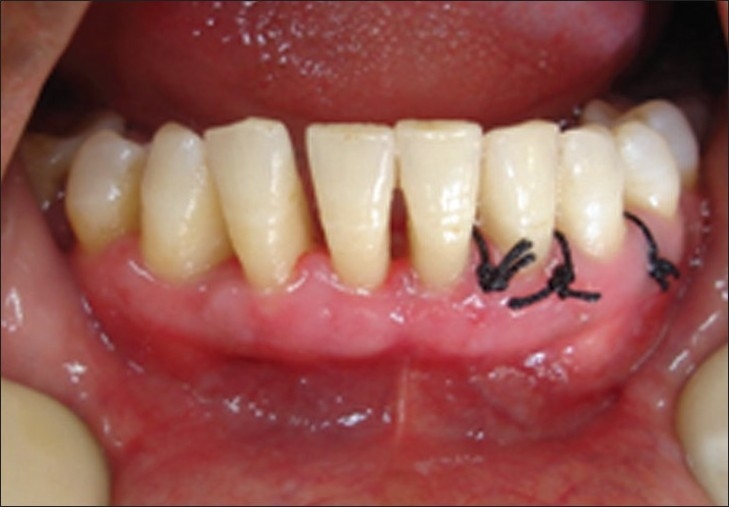
After 3 days, case 2

**Figure 11:**
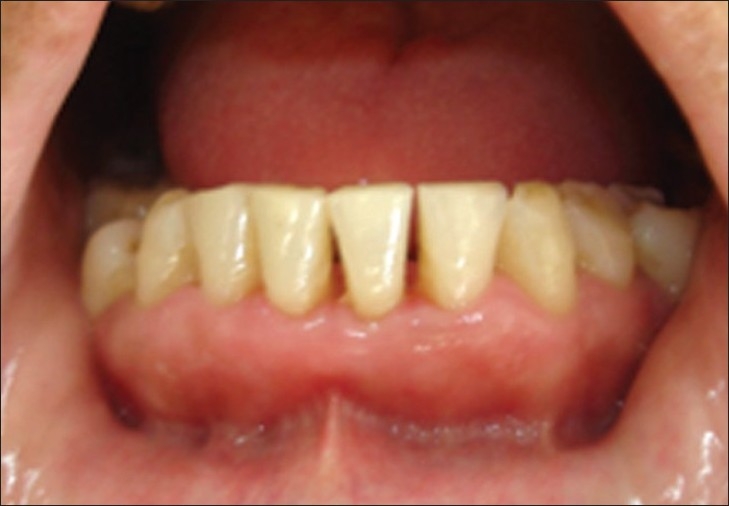
After seven days, case 1

**Figure 12:**
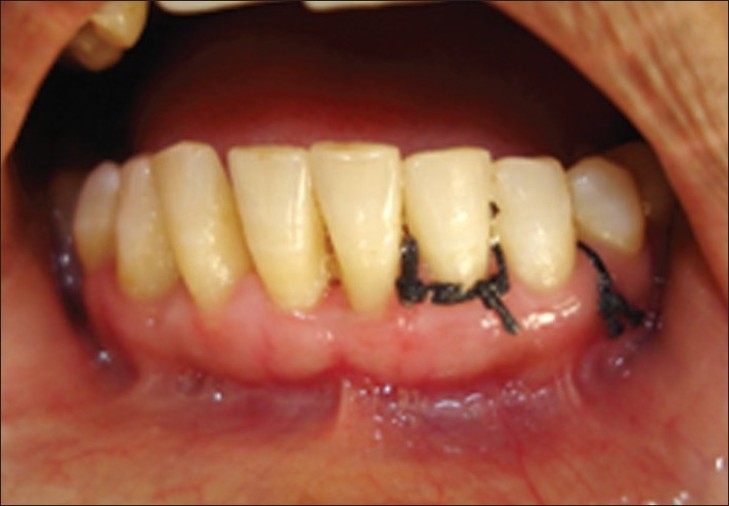
After seven days, case 2

**Figure 13:**
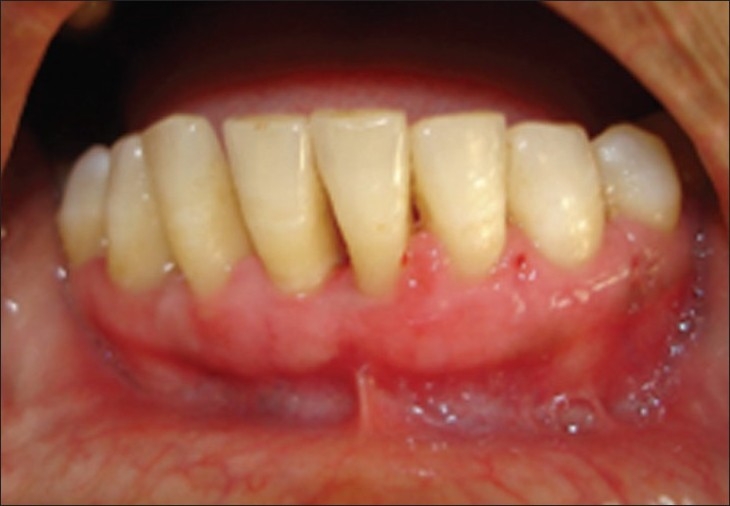
After seven days, case 2, plaque retention below sutures. At the time of suture removal bleeding points are clearly seen at the site of needle insertion.

**Figure 14:**
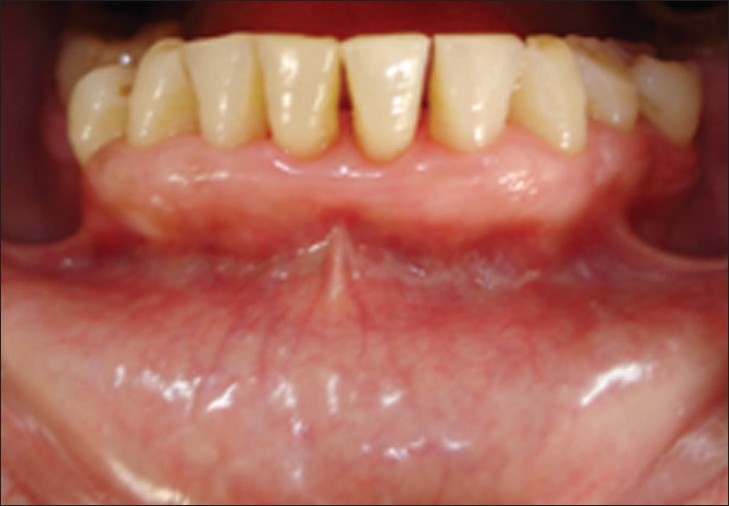
After 20 days, case 1

**Figure 15:**
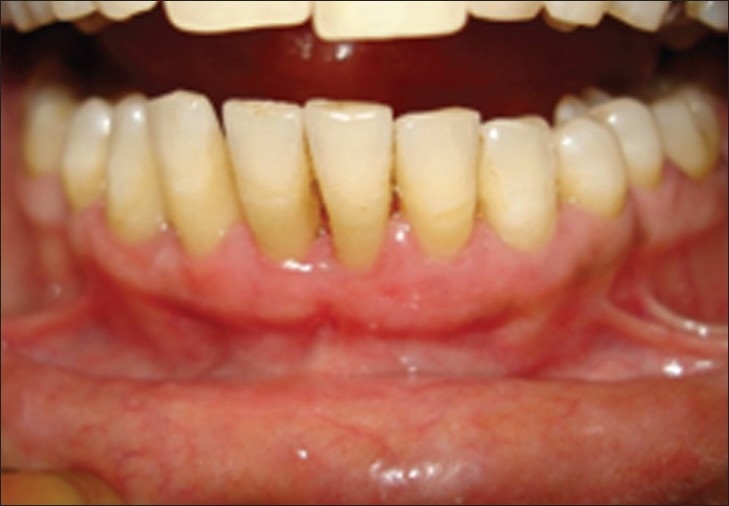
After 20 days, case 2

**Figure 16:**
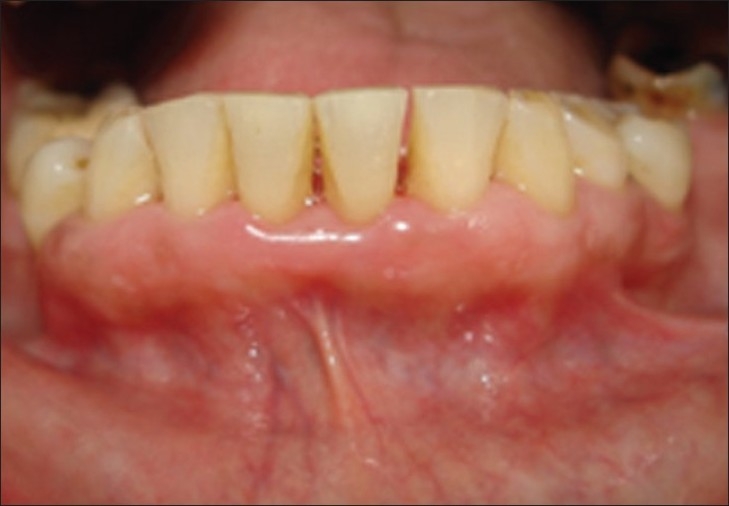
After three months, case 1

**Figure 17:**
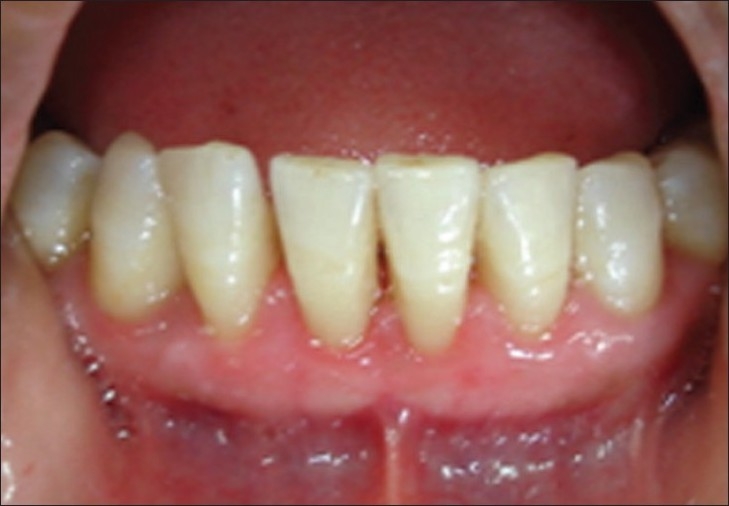
After three months, case 2

## DISCUSSION

It is worth noting that the overall convenience in using fibrin sealing system should be evaluated on the basis of its cost and benefits.

To evaluate fibrin sealing system in the clinical trial, Ethicon Mersilk 3–0 sutures were used as a control since the latter is the usual means of fixing tissues in periodontal surgery. Synthetic adhesives (cyanoacrylates) have been discarded because of toxicity, stiffness, and lack of acceptance by clinicians. Resorbable sutures use sometimes known to cause strong inflammatory responses.

It appeared that the essential precursor for connective tissue attachment was occurrence of fibrin linkage with the root surface, and this attachment became replaced by collagen to establish new connective tissue attachment to a denuded root surface.

However from biologic stand point, the fibrin glue seemed both innocuous and effective, also bringing about early wound healing. Fibronectin, a family of related proteins found in blood plasma and on fibroblast surfaces is a chemoattractant for fibroblasts and enhances the interaction and adherence of fibroblast to surfaces.

Fibronectin may serve to anchor a blood clot to surrounding collagen owing to its property of being covalently linked to fibrin and collagen by factor XIII a. Both hemostatic and adhering effects can be modulated using thrombin in different concentrations: 4NIH thrombin allows 20–30 seconds to adjust flaps or grafts before clotting, 500NIH solution reduces operative and clotting times, especially useful for small flaps since positioning of tissues must be accomplished less than 5–10 seconds.

Conventional sutures provides only a marginal fixation, while the fibrin sealing system makes the tissues adhere on its whole surface.

Fibrin glue is a cryo precipitate of human plasma, but vapor heating during manufacturing prevents any risk for transmission of AIDS or infectious hepatitis.

The use of fibrin glue saves remarkable amount of time and makes it easier to fix the tissues in difficult inaccessible areas and esthetically critical areas. The time saved range from 3–19.5 minutes per procedure, 1–8.5 minutes per tooth.

Fibrin sealing system also saves cost of preparing, sterilizing, and storing instruments at the time of suture removal as well as cost of sutures themselves.

Further advantages on patient's side: removal of sutures at times is annoying or painful for some patients, especially children. There is a comfort feel on sutureless side in the days after surgery.

## CONCLUSION

1. The fibrin glue is easier and quicker to use than sutures.
2. The fibrin sealing system provides better early hemostasis and complete adhesion of the whole surface of the tissues to the underlying layer.
3. Sutures cause inflammation themselves; fibrin glue enhances early wound healing.
4. The fibrin sealing system is effective as a means of fixing tissues after periodontal surgery
